# Chronic Red Bull Consumption during Adolescence: Effect on Mesocortical and Mesolimbic Dopamine Transmission and Cardiovascular System in Adult Rats

**DOI:** 10.3390/ph14070609

**Published:** 2021-06-24

**Authors:** Romina Vargiu, Francesca Broccia, Carla Lobina, Daniele Lecca, Alessandro Capra, Pier Paolo Bassareo, Valentina Bassareo

**Affiliations:** 1 Department of Biomedical Sciences, University of Cagliari, Cittadella Universitaria SP 8, Km 0.700, 09042 Monserrato, Italy; francesca.broccia@gmail.com (F.B.); dlecca@unica.it (D.L.); acapra@unica.it (A.C.); 2 Neuroscience Institute, National Research Council of Italy, Section of Cagliari, Cittadella Universitaria SP 8, Km 0.700, 09042 Monserrato, Italy; carlalobina@hotmail.com; 3 Department of Cardiology, University College of Dublin, Mater Misericordiae University Hospital, D07 R2WY Dublin, Ireland; piercard@inwind.it

**Keywords:** energy drinks, dopamine, nucleus accumbens, prefrontal cortex, cardiovascular hemodynamic indices, cardiac contractility

## Abstract

Energy drinks are very popular nonalcoholic beverages among adolescents and young adults for their stimulant effects. Our study aimed to investigate the effect of repeated intraoral Red Bull (RB) infusion on dopamine transmission in the nucleus accumbens shell and core and in the medial prefrontal cortex and on cardiac contractility in adult rats exposed to chronic RB consumption. Rats were subjected to 4 weeks of RB voluntary consumption from adolescence to adulthood. Monitoring of in vivo dopamine was carried out by brain microdialysis. In vitro cardiac contractility was studied on biomechanical properties of isolated left-ventricular papillary muscle. The main finding of the study was that, in treated animals, RB increased shell dopamine via a nonadaptive mechanism, a pattern similar to that of drugs of abuse. No changes in isometric and isotonic mechanical parameters were associated with chronic RB consumption. However, a prolonged time to peak tension and half-time of relaxation and a slower peak rate of tension fall were observed in RB-treated rats. It is likely that RB treatment affects left-ventricular papillary muscle contraction. The neurochemical results here obtained can explain the addictive properties of RB, while the cardiovascular investigation findings suggest a hidden papillary contractility impairment.

## 1. Introduction

Energy drinks (EDs) appeared for the first time in Europe and Asia in the 1960s, but their popularity grew when Red Bull (RB) was introduced in Austria in 1987 [1]. The use and abuse of EDs are increasing continuously worldwide [2,3], and they are consumed frequently by young people aged between 13 and 35 for their psychoactive, stimulant, and performance-enhancing properties. The feeling of power and the absence of fatigue are often confused with euphoria, promoting the excessive consumption of these beverages in adolescents. For the youngest among them, this is one of the first transgressions in their lives.

The stimulant effects are mostly due to the high concentration of caffeine and sucrose and to the presence of taurine, but various B vitamins, such as nicotinamide, pyridoxine, and riboflavin, as well as ginseng and guarana seed extract, are also present [4,5]. ED consumption is increasing constantly, and the consequent negative effects on the central nervous system (CNS) and cardiovascular system due to excessive caffeine and taurine are also on the rise, above all, among, adolescents [6,7]. Several articles reported the incidence of ED consumption and the occurrence of cerebral vasculopathy [8], acute mania [9,10], epileptic seizures [5,11], coronary artery vasospasm [12], and severe cardiac arrest or myocardial ischemia [13,14]. The American Journal of Emergency Medicine [15] illustrated a case report of a healthy adult man who had ischemic stroke and epileptic seizures after consuming EDs with alcohol. Moreover, a negative hemodynamic profile was observed after acute RB ingestion in young adults. In particular, the authors found an increase in both systolic and diastolic blood pressure, associated with an increase in heart rate, cardiac output, cerebrovascular resistance, and breathing frequency, as well as a decrease in cerebral blood flow velocity and end-tidal carbon dioxide [16]. Basrai et al. [17], in a randomized crossover trial, demonstrated that EDs induce acute cardiovascular and metabolic changes, posing potential risks to young adults.

The effects of EDs on the cardiovascular system and CNS have also been reported in several animal studies. Ebuehi et al. [18] showed that, in rabbits, oral administration of EDs, such as power horse and RB, affected blood chemistry and liver enzyme activities but did not change the histopathology of the brain, heart, and liver. Other authors reported that, in adult rats, chronic consumption of RB alone or in association with ethanol produced biochemical and ultrastructural alterations in the heart muscle [19]. Moreover, Diaz et al. [20] observed that chronic consumption of alcohol in combination with EDs caused an inflammatory response and oxidative stress, which induced cell death via apoptosis in the hippocampus and temporal cortex of adult rats.

Unfortunately, a lack of literature exists on the effect of EDs on neurotransmission in the CNS, particularly in the rewarding brain circuits. These circuits, including the dopaminergic mesolimbic system, are involved in the response to rewarding stimuli, both natural (food, sex, maternal care, etc.) and pharmacological (drugs of abuse) [21]. In particular, it has been shown that the release of the neurotransmitter dopamine (DA) is preferentially increased in an important area of this system, the nucleus accumbens (NAc) shell, after the consumption of both food [22,23,24,25,26,27,28,29,30,31] and drugs of abuse [25,32,33,34,35,36,37,38,39], but with a different pattern. In fact, while the increase in DA in the shell is inhibited after the first food consumption [22,24,27,28,40], the repeated administration of drugs of abuse repeatedly stimulates DA in this area [25,35,36,37,38,41]. It has been hypothesized that the repeated stimulation of DA transmission in the NAc shell by repeated administration of drugs of abuse could be one of the mechanisms that underlie drug addiction [42,43].

Furthermore, the effect of caffeine, one of the main components of all EDs, on mesolimbic DA is still debated. Previous research in our laboratory demonstrated that intraperitoneal injection of caffeine did not increase the DA concentration in the NAc shell [44,45]. In contrast, other groups observed stimulation of DA transmission in the same area [46,47]. Moreover, a significant increase in extracellular DA in the NAc shell was observed after exposure to volatilized caffeine [48].

With the aim of clarifying the potential for abuse of RB, we investigated, by “in vivo” microdialysis, the effect of its repeated oral administration on the responsiveness of mesolimbic and mesocortical DA transmission in adult rats subjected to chronic RB consumption from adolescence to adulthood.

With the additional goal of studying the effects of chronic RB consumption on cardiac contractility, at the end of the microdialysis experiments, we investigated the left-ventricular papillary muscle contractile activity of isolated hearts from RB-treated rats. Furthermore, heart rate and blood pressure were recorded weekly in order to evaluate the impact of chronic RB consumption on cardiovascular hemodynamic parameters. During the study, daily amounts of water and RB were monitored, as well as weekly amounts of food, animal weight, and glycemia, with the purpose of establishing whether chronic consumption of ED can affect these parameters.

The neurochemical experiments will be determinant for knowledge of whether RB stimulates DA transmission in the NAc shell and core, and in the PFCX with a pattern similar to drugs of abuse or to food. The results obtained could let us to justify the rewarding property of RB and its compulsive consumption. The findings of our study of the effects of chronic RB consumption during adolescence on cardiac contractility, heart rate, and blood pressure could highlight a higher probability of onset of cardiomyopathy in adulthood.

## 2. Results

### 2.1. Consummatory and Metabolic Data

#### 2.1.1. Fluid Intake in Control and Treated Rats during the Dark and the Light Period of the R Week and the 4 Weeks of Treatment

**Figure 1:**
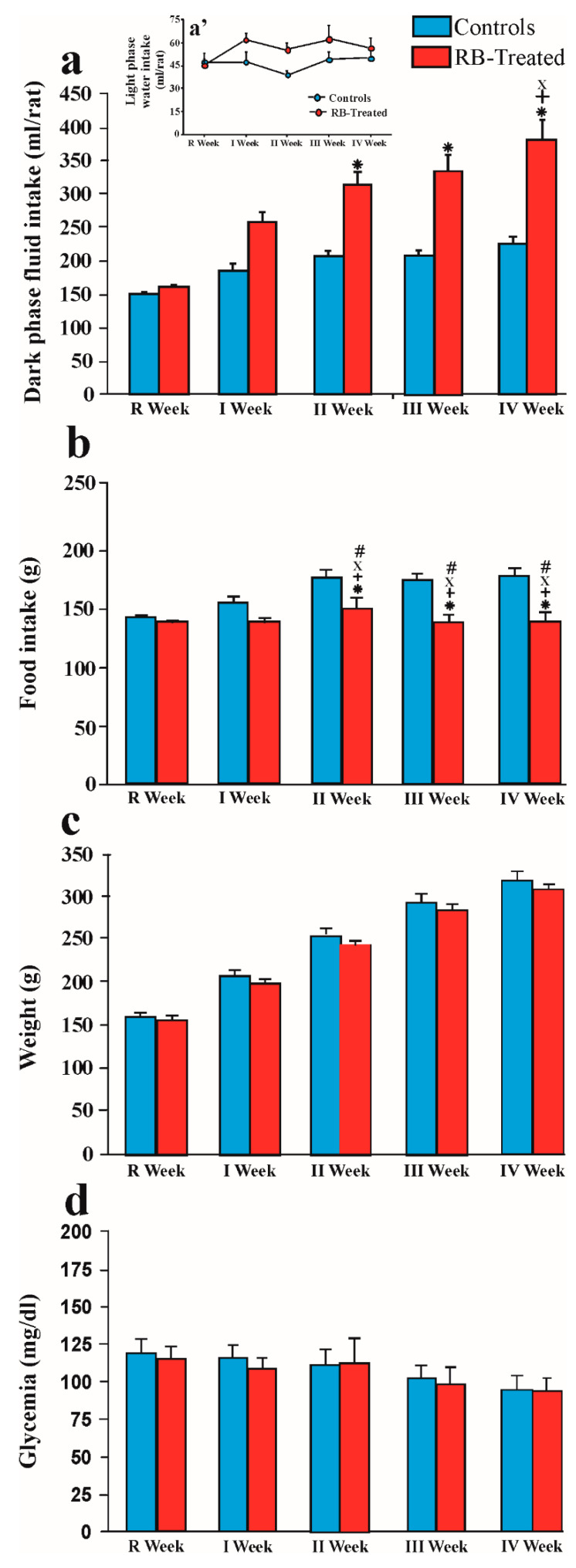
Consummatory and metabolic data during the R week and the 4 weeks of treatment. (**a**) Dark phase fluid intake in control and RB-treated rats. *p* < 0.001 vs. control rats: * I Week; + II Week; × III Week. (Insert **a’**) Light phase water intake in control and RB-treated rats. (**b**) Amount of food eaten by control and RB-treated rats. *p* < 0.01 vs. controls * I Week; + II Week; × III Week; # IV Week. (**c**) Weight of control and RB-treated rats. (**d**) Levels of fasting blood sugar in control and RB-treated rats. Statistical analysis: two-way ANOVA.

Figure 1a shows the amount of water consumed during the dark phase of the R week by both groups. One-way ANOVA did not show any differences between groups (F1,30 = 3.6; *p* = 0.06).

The figure also shows the weekly amount, expressed in mL/rat, of water or RB consumed by control and treated rats, respectively, during the dark phase of the 4 weeks of treatment.

Two-way ANOVA showed a significant effect of group (F1,30 = 33.1; *p* < 0.001), time (F4,120 = 40.58; *p* < 0.001), and the group × time interaction (F4,120 = 15.38; *p* < 0.001). The post hoc test revealed that the amount of RB taken by treated rats increased significantly during RB treatment and was greater than the amount of water taken by control animals.

Figure 1a’ shows the weekly amount, expressed in mL/rat, of water consumed by control and treated rats, respectively, during the light phase of the R week and the 4 weeks of treatment.

Two-way ANOVA did not show any differences between groups (Fgroup1,30 = 3.54; *p* = 0.07; Ftime4,120 = 1.61; *p* = 0.18; Fgroup×time4,120 = 1.23; *p* = 0.3).

The total 24 h of fluid consumption is reported in Table 1.

Two-way ANOVA showed a significant effect of group (F1,30 = 33.6; *p* < 0.001), time (F4,120 = 41.64; *p* < 0.001), and the group × time interaction (F4,120 = 10.77; *p* < 0.001).

#### 2.1.2. Amount of Food Eaten by Control and RB-Treated Rats during the R Week and the 4 Weeks of Treatment

Figure 1b shows the weekly amount, expressed in g/rat, of food eaten by control and RB-treated animals during the R week and during the 4 weeks of treatment. Two-way ANOVA showed a significant effect of group (F1,14 = 12.65; *p* = 0.003), time (F4,56 = 4.76; *p* = 0.002), and the group × time interaction (F4,56 = 7.52; *p* = 0.001). Tukey’s post hoc test revealed that the amount of food eaten by RB-treated rats was significantly decreased compared with the amount of food eaten by control animals.

#### 2.1.3. Weight of Control and RB-Treated Rats during the R Week and the 4 Weeks of Treatment

Figure 1c shows the weekly weight of control and RB-treated rats, expressed in grams, during the R week and during the 4 weeks of treatment. Two-way ANOVA showed a significant effect of time (F4,56 = 1121.92; *p* < 0.001). The post hoc test revealed that the animals’ weights in the two groups were comparable.

#### 2.1.4. Levels of Fasting Blood Sugar in Control and RB-Treated Rats during the R Week and the 4 Weeks of Treatment

Figure 1d shows the levels of fasting blood sugar in control and RB-treated rats during the R week and during the 4 weeks of treatment. Two-way ANOVA showed a significant effect of time (F4,56 = 16.21; *p* < 0.001). The post hoc test did not show any difference between the levels of fasting blood sugar in the two groups measured in the same week.

### 2.2. Energy Intake by RB-Treated and Control Rats during the 4 Weeks of Treatment

The quantities of individual active ingredients contained in the RB consumed by RB-treated rats in each week of treatment and the corresponding energy intake are shown in Table 2.

**Figure 2:**
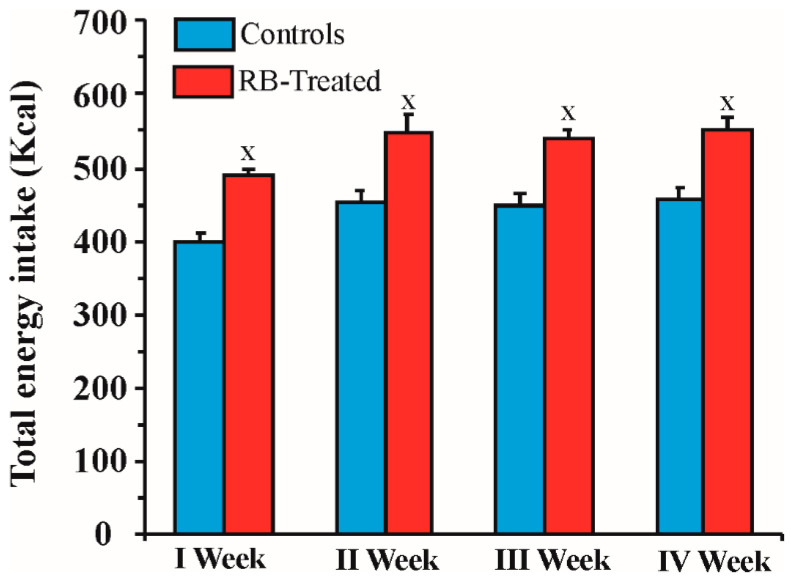
Total energy intake in control and RB-treated rats during the 4 weeks of treatment. Values are expressed as the mean ± SEM. × *p* < 0.001 RB-treated vs. controls. Statistical analysis: two-way ANOVA.

Figure 2 shows the weekly total energy intake in control (food eaten) and RB-treated (food eaten + RB) animals during the 4 weeks of treatment. Two-way ANOVA showed a significant effect of treatment (F1,14 = 20.95; *p* = 0.0004) and time (F3,42 = 13.99; *p* < 0.0001). Post hoc analysis by Sidak’s multiple comparisons revealed that the caloric input in RB-treated rats was significantly increased compared with control ones in all weeks of treatment (*p* < 0.001).

### 2.3. Effect of Chronic Red Bull Consumption on Blood Pressure and Heart Rate

#### 2.3.1. SBP

**Figure 3:**
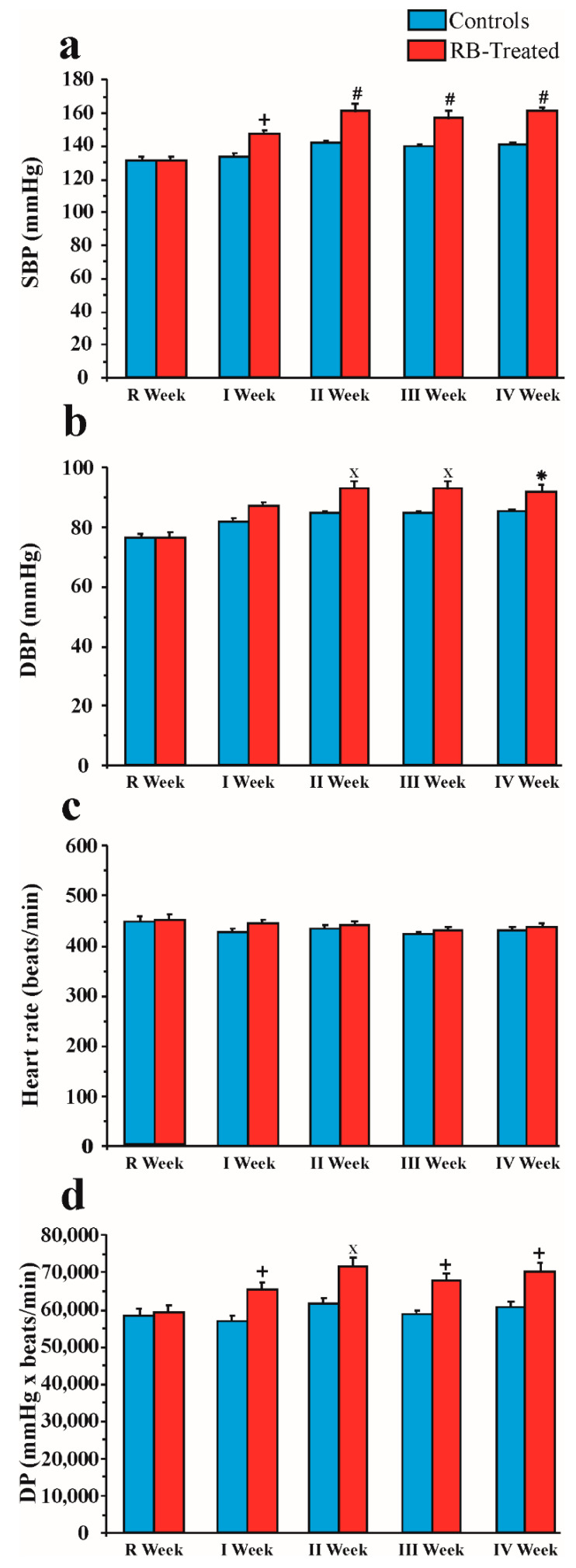
Cardiovascular hemodynamic parameters in RB-treated and control rats during the 4 weeks of treatment. (**a**) SBP, systolic blood pressure; (**b**) DBP, diastolic blood pressure; (**c**) heart rate; (**d**) DP, double product. Values are expressed as the mean ± SEM; RB-treated vs. controls: * *p* < 0.05, + *p* < 0.005, × *p* < 0.001, # *p* < 0.0001. Statistical analysis: two-way ANOVA.

Figure 3a shows SBP obtained in control and RB-treated rats. Two-way ANOVA revealed a significant effect of RB treatment (F1,30 = 37.71; *p* < 0.0001), time (F4,120 = 30.05; *p* < 0.0001), and treatment × time interaction (F4,120 = 6.55; *p* < 0.0001). Post hoc analysis by Sidak’s multiple comparisons test revealed a significant enhancement of SBP in the RB-treated group compared with the control group in all weeks of treatment (I week, *p* < 0.005; II, III, and IV weeks, *p* < 0.0001).

#### 2.3.2. DBP

Figure 3b shows DBP obtained in control and RB-treated rats. Two-way ANOVA highlighted significant effects of RB treatment (F1,30 = 21.01; *p* < 0.0001), time (F4,120 = 27.12 *p* < 0.0001), and treatment × time interaction (F4,120 = 2.98; *p* < 0.05). Post hoc analysis by Sidak’s multiple comparisons revealed a significant increase of DBP in the RB-treated group in II and III weeks (*p* < 0.001) and in IV week (*p* < 0.05).

#### 2.3.3. Heart Rate

Figure 3c shows the heart rates observed in control and RB-treated rats. Two-way ANOVA revealed no significant effect of RB treatment (F1,30 = 1.22; *p* > 0.05) on heart rate values, a significant effect of time (F4,120 = 2.48; *p* < 0.05), and no significant treatment × time interaction (F4,120 = 0.39; *p* > 0.05).

#### 2.3.4. DP

Figure 3d shows DP calculated in control and RB-treated rats. Two-way ANOVA revealed a significant effect of RB treatment (F1,150 = 44.75 *p* < 0.0001) and weeks (F4,150 = 6.47; *p* < 0.0001), but no significant treatment × time interaction (F4,150 = 2.37; *p* > 0.05). Post hoc analysis by Sidak’s multiple comparisons test showed a significant increase of DP in the RB-treated group compared with the control group in all weeks of treatment (I week, *p* < 0.005; II week, *p* < 0.001; III and IV weeks, *p* < 0.005).

### 2.4. Microdialysis and Taste Reactivity Experiments

#### 2.4.1. NAc Shell and Core DA Responsiveness and Taste Reactions after Repeated Intraoral RB Administration in Treated and Control Animals

**Figure 4:**
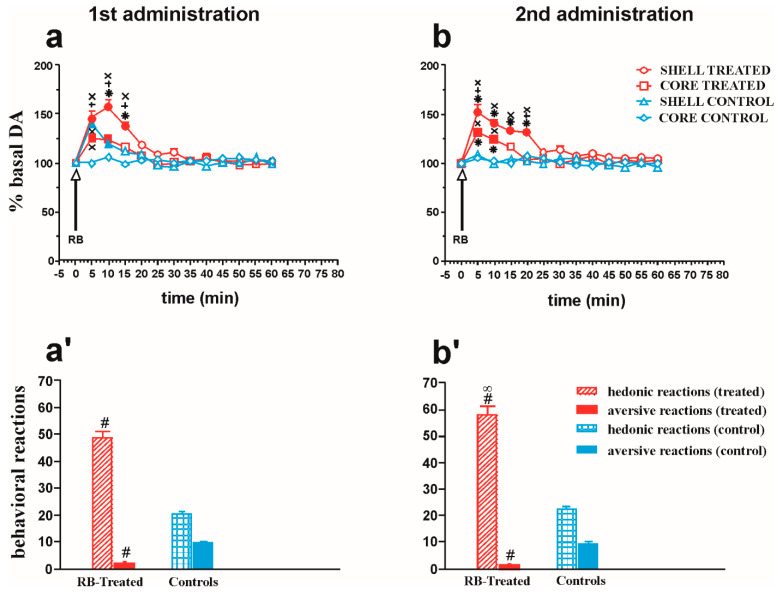
Changes in NAc shell and core dialysate DA after two subsequent intraoral administrations of RB (2 mL) in RB-treated and control rats. Figure 4 also shows the score of hedonic and aversive taste reactions during intraoral RB infusion. (**a**,**b**) DA transmission: filled symbols *p* < 0.001 vs. basal value; * *p* < 0.05 vs. DA responsiveness in the shell of control rats; + *p* < 0.05 vs. DA responsiveness in the core of RB-treated rats; × *p* < 0.05 vs. DA responsiveness in the core of control rats. (**a’**,**b’**). Statistical analysis: four-way ANOVA. Taste reactions: # *p* < 0.001 vs. control rats; ∞ *p* < 0.001 vs. first administration. Statistical analysis: two-way ANOVA.

Figure 4a,b show the changes in NAc shell and core dialysate DA after two subsequent intraoral administrations of RB (2 mL) in RB-treated and control rats. Figure 4a’,b’ show the score of hedonic and aversive taste reactions during intraoral RB infusion.

Four-way ANOVA showed a significant effect of RB treatment (F1,32 = 70.51; *p* < 0.001), area (F1,32 = 31.08; *p* = 0.001), time (F12,384 = 59.84; *p* < 0.001), and RB treatment × RB pre-exposure interaction (F1,32 = 5.27; *p* = 0.03). Moreover, significant differences were detected in the interactions of RB treatment × area (F1,32 = 13.85; *p* < 0.01), RB treatment × time (F12,384 = 21.30; *p* < 0.001), RB pre-exposure × time (F12,384 = 2.41; *p* = 0.005), area × time (F12,384 = 10.59; *p* < 0.001), RB treatment × area × time (F12,384 = 2.6; *p* = 0.002), RB pre-exposure × area × time (F12,384 = 3.43; *p* < 0.001), and RB treatment × RB pre-exposure × area × time (F12,384 = 2.19; *p* = 0.01).

Tukey’s post hoc test revealed that, during the first RB exposure, DA increased to a higher extent in the shell of RB-treated than in the core of RB-TREATED rats and the NAc shell of control rats. Furthermore, during the second RB exposure, the increase of DA was higher in the NAc shell of treated than in the core of RB-treated rats and the shell of control rats.

A preliminary analysis of hedonic or aversive reactions from animals who had a microdialysis probe in the shell or in the core was conducted and showed no effect of area on the reaction; hence, the results from these two groups were combined into one.

Two-way ANOVA of hedonic reactions showed a significant effect of RB treatment (F1,18 = 152.6; *p* < 0.001) and RB pre-exposure (F1,18 = 9.94; *p* = 0.05). The post hoc test revealed that hedonic reactions in RB-treated rats were stronger than those in control rats. Two-way ANOVA of aversive reactions showed a significant effect of RB treatment (F1,18 = 216.45; *p* < 0.001). The post hoc test revealed stronger aversive reactions in control rats than in treated rats.

#### 2.4.2. Effect of Repeated Intraoral RB Infusion on DA Transmission in the Medial PFCX and on Taste Reactions

**Figure 5:**
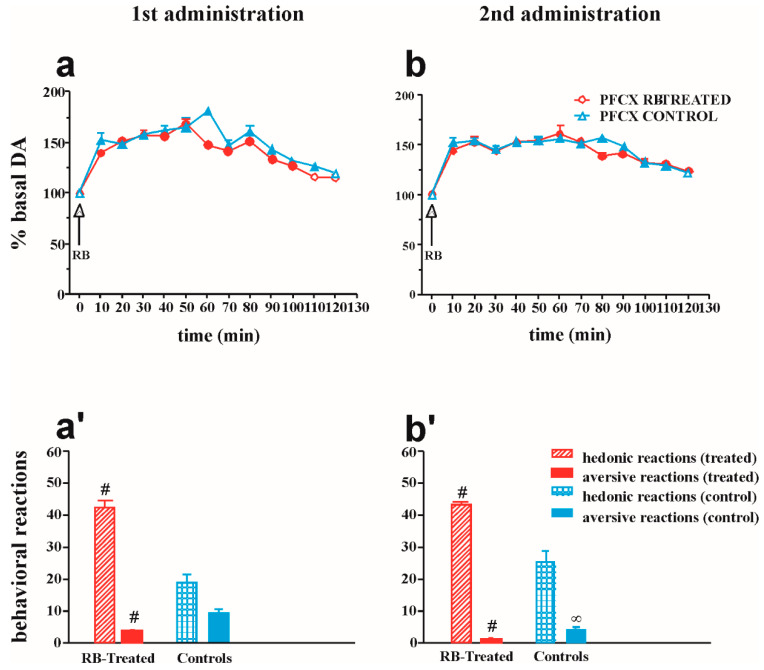
mPFCX DA responsiveness and taste reactions after repeated intraoral RB administration in RB-treated and control animals. Figure 5 also shows the score of hedonic and aversive taste reactions during intraoral RB infusion. (**a**,**b**) DA transmission: filled symbols *p* < 0.001 vs. basal value. (**a’**,**b’**). Statistical analysis: three-way ANOVA. Taste reactions: # *p* < 0.001 vs. control rats, ∞ *p* < 0.001 vs. first administration. Statistical analysis: two-way ANOVA.

Figure 5a,b show the changes in mPFCX dialysate DA after two subsequent intraoral administrations of RB (2 mL) in RB-treated and control rats. Figure 5a’,b’ show the score for hedonic and aversive taste reactions during intraoral RB infusion.

Three-way ANOVA showed a significant effect of RB treatment (F1,20 = 11.48; *p* = 0.003), time (F12,24 = 89.26; *p* < 0.001), interaction of RB treatment × time (F12,24 = 2.15; *p* = 0.01), and interaction of RB treatment × RB pre-exposure × time (F12,24 = 2.34; *p* = 0.007). Tukey’s post hoc test revealed an increase of PFCX DA in both RB-treated and control animals during the first and second RB administrations.

Two-way ANOVA of hedonic reactions showed a significant effect of RB treatment (F1,10 = 51.14; *p* < 0.001), and the post hoc test revealed that hedonic reactions in RB-treated rats were stronger than in control rats. Two-way ANOVA of aversive reactions showed a significant effect of RB treatment (F1,10 = 21.81; *p* < 0.001) and RB pre-exposure (F1,10 = 19.36; *p* = 0.001). The post hoc test revealed stronger aversive reactions in control rats than in RB-treated rats.

### 2.5. Physiological “In Vitro” Study

Table 3 shows comparisons of the isometric and isotonic parameters between control and RB-treated rats. Unexpectedly, RB consumption did not result in a significant change in the following isometric and isotonic mechanical experimental parameters of left-ventricular PMs: RT and P_0_, and ΔL and Vmax (*p* > 0.05).

**Figure 6:**
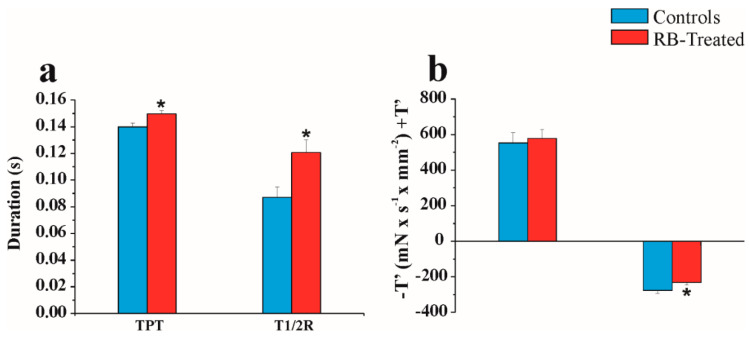
Isometric contractility indices in controls and RB-treated LVPMs. (**a**) TPT, time to peak tension, and T ½ R, half-time of relaxation. (**b**) +T’, peak rate of tension rise, and −T’, peak rate of tension fall. Values are expressed as the mean ± SEM; * *p* < 0.05. Statistical analysis: Student’s *t*-test.

Figure 6 reports the most striking differences of isometric timing parameters (a) and +T’ and −T’ (b), recorded at Lmax and 0.06 Hz, of control and RB-treated left-ventricular PMs. As shown in Figure 6, Student’s *t*-test reported that (a) TPT and T½R were significantly prolonged in RB-treated PM compared with control rats (*p* < 0.05), whereby TPT was 0.149 ± 0.002 vs. 0.139 ± 0.002 s, and T½R was 0.120 ± 0.009 vs. 0.087 ± 0.007 s in RB-treated and controls, respectively, and (b) the peak rate of tension fall, −T’, was significantly slower (*p* < 0.05) in RB-treated compared with control left-ventricular PMs, whereby −T’ was 231.79 ± 14.24 vs. 276.51 ± 17.50 mN × s^−1^ × mm^2^, in RB-treated and controls, respectively.

While +T’ remained unchanged, all the other parameters, TPT, T ½ R, and −T’, were abnormal in RB-treated compared with controls, suggesting a hidden papillary contractility impairment.

The application of the Huxley’s mathematical formalism did not highlight significant differences in the mechanics and energetics of myosin molecular motors of the LVPMs of RB-treated animals compared with control ones (data not shown).

## 3. Discussion

The main finding of the present study is that chronic administration of large quantities of RB during adolescence increases the DA release in the NAc shell after repeated administration, showing a nonadaptive mechanism in the stimulation of DA transmission similar to that observed after drugs of abuse administration.

It is well known that drugs of abuse, independently of their mechanism of action, stimulate DA transmission in the mesolimbic system, specifically in the NAc shell, after either contingent [35,36,37,38,41] or noncontingent administration [25,32,33,34,39]. The NAc shell is part of the rewarding brain circuits, and DA in this area responds with a different pattern to several rewarding stimuli, such as pharmacological (drugs of abuse) and natural (food) rewards. In fact, while the increase in DA in the shell undergoes rapid habituation after the first food consumption [22,24,27,28,40], repeated administration of drugs of abuse continuously stimulates DA in the shell [25,35,36,37,38,41]. The adaptive mechanism (habituation) shown by shell DA responsiveness to food supports its hypothesized facilitatory role in the establishment of associations between the rewarding properties of food and secondary stimuli that acquire predictive value. The consequence of nonadaptive stimulation of DA release in the NAc shell is an abnormal and pathological associative learning that seems to be one of the mechanisms that underlie drug addiction [42,43].

In our research, we studied the DA responsiveness to repeated administration of one of the most popular EDs, RB. EDs are consumed for their psychoactive, performance-enhancing, and stimulant properties, which are mostly due to the high concentration of caffeine and sucrose and to the presence of taurine.

Caffeine is an antagonist of A2A adenosine receptors with psychostimulant properties, and its effect on mesolimbic DA is still debated. Previous research from our laboratory demonstrated that i.p. injection of caffeine did not increase DA concentration in the NAc shell [44,45]. In contrast, other groups observed a stimulation of DA transmission in this area [46,47]. A significant increase in the extracellular DA in the NAc shell was observed after exposure to volatilized caffeine [48].

It is well known that sugar stimulates DA transmission in the NAc shell [27,29,30,31,49,50], and several authors reported that this DA response undergoes habituation [27,29]. Only continuous or intermittent sugar intake or the operant behavior for sucrose impairs the habituation phenomenon [27,29,30,31,51,52]. Moreover, few studies exist on the effect of taurine, the third important component of RB, on NAc DA transmission. It has been shown that local intra-NAc infusions of taurine at different concentrations increased DA release in this area, and that this effect was mediated by strychnine-sensitive glycine receptors [53].

To our knowledge, this is the first “in vivo” study on the effect of an ED containing high levels of caffeine, sucrose, and taurine on DA transmission in the NAc shell and core and in the mPFCX. In particular, chronic RB administration was performed in adolescent rats, and the long-lasting effects on the brain and cardiovascular system were evaluated in adult animals, in order to replicate as closely as possible the modality of ED intake observed in humans.

We found that chronic RB consumption during adolescence could modify the pattern of DA responsiveness after acute RB oral infusion. In fact, while in controls the first RB administration increased extracellular DA in the NAc shell, the second, 2 h later, was unable to do that, and DA responsiveness underwent habituation. This pattern of DA responsiveness in the NAc shell is typical of natural rewards such as food, and it has been reported by many authors [22,24,27,28,40]. However, in RB-treated rats, both the first and the second RB administration affected DA transmission in the NAc shell, showing a pattern similar to that observed after repeated drugs of abuse administration. We also found a stimulation of DA transmission in the NAc core during both the first and the second RB infusion.

As expected, DA transmission was stimulated in the mPFCX during both subsequent RB infusions in both RB-treated and control rats, showing a long-lasting increase. This is not surprising, as it is well known that mPFCX DA transmission is affected by food consumption [22] and caffeine administration [44,45]. The results obtained by in vivo microdialysis led us to hypothesize that the repeated increase in DA in the NAc shell after repeated RB administrations in treated rats could play a key role in the potential abuse of this ED.

The abuse or excessive consumption of EDs is constantly increasing on a global scale, and children, adolescents, and young adults are the major consumers [2,3,54,55,56]. ED intake by these users may have potential negative side-effects on their health, such as tachycardia, hypertension, confusion, agitation, seizures, liver damage, kidney failure, and cardiac dysfunction [57,58]. Moreover, another point of concern is that adolescents who frequently consume EDs have a higher risk of alcohol abuse and use of illegal drugs [59,60,61,62], as well as a lower perceived risk in using them [63]. These studies strengthen the argument for a role of EDs in the gateway hypothesis, which requires further detailed study.

In our protocol, RB-treated animals consumed a large amount of RB (380 mL in the last week), higher than the volume of water consumed by controls (225 mL in the last week). These results led us to hypothesize a preference for the taste of RB, probably due to its palatability and rewarding effects. As shown by taste reactivity analysis, RB-naïve rats revealed stronger aversive taste reactions, during both the first and the second intraoral RB infusions, compared with RB-treated animals. In contrast, RB-treated rats revealed stronger hedonic reactions during both the first and the second RB intraoral infusions with respect to controls. These observations led us to conclude that prolonged RB treatment during the adolescence positively modified the taste RB perceived by animals, increasing the hedonic value of the ED.

Furthermore, during our study, the weekly amount of food eaten, animal weights, and glycemia were monitored in order to verify whether RB intake was able to modify these parameters. From the metabolic point of view, the findings obtained indicate that RB treatment induces a lower consumption of food in treated rats than in control ones. On the other hand, considering the caloric intake derived from the consumption of RB, the treated rats received a weekly caloric supplement of 20% compared with controls. Nevertheless, throughout the treatment, there was no difference in body weight gained between the two groups. Therefore, the supplemental energy was not stored in the endogenous lipid reserves of RB-treated animals but was readily used. To our knowledge, these results could be associated with the effects of caffeine contained in the RB. It has been demonstrated that caffeine induces negative energy balance by altering multiple aspects of behavior and metabolism, including physical activity, thermogenesis, and energy expenditure [64].

Caffeine alone is known to induce a thermogenic effect and to increase resting energy expenditure in an animal model [65]. In addition, it has been shown that caffeine promotes fatty-acid oxidation; therefore, the latter effect and/or its thermogenic properties could be responsible for weight management [66,67,68,69,70].

Furthermore, in order to assess the cardiovascular influence of the ED RB, we performed weekly hemodynamic measurements in each animal. Subsequently, we investigated the effects of chronic RB consumption on cardiac contractility.

Several studies have shown alterations in hemodynamic parameters and cardiac function in response to ED consumption in humans [14,17,71], with a few in animal models [19]. In keeping with these observations, our data indicated that chronic RB consumption in rats maintained at rest had no effect on HR and increased SBP, DBP, and DP, indicating an increased cardiac workload. These findings are in agreement with previous observations regarding the effects of caffeine and/or EDs on BP and resting heart rate [72,73,74]. The correlation of caffeinated beverage consumption with blood pressure in adolescents was demonstrated by Savoca et al. [75,76], who found an association between caffeine intake and increased SBP and DBP in African Americans.

Interestingly, it has been reported that, in young, healthy adults, acute RB consumption increased BP and DP at rest, as well as HR and cardiac output [16], and that these effects occur through different hemodynamic pathways than those used by a comparable amount of caffeine [71]. This hypothesis is supported by Pincomb et al. [77] and Sung et al. [78], who observed an alteration in hemodynamic parameters and an increase in peripheral vascular resistance but no change in cardiac contractility after 3.3 mg/kg body weight of caffeine consumption in healthy young men. Therefore, it would seem that the effects of RB were myocardial, while caffeine elicited vascular effects.

However, in our “in vitro” experimental findings, there was no evidence of enhanced cardiac contractility. Rather, biomechanical characteristics, such as RT, P_0_, DL, and Vmax of LVPM of rats treated with the ED RB did not reveal any significant differences compared with control ones. On the other hand, the timing parameters of contraction and relaxation, TPT, T½R, and −T’, were longer and slower, respectively, in the papillary muscles of RB-treated animals than in control ones. It would seem from this evidence that long-term RB treatment induced early and hidden papillary contractility impairment.

Our functional observations could be explained by an original investigation [19] that showed long-term effects of RB intake, individually or in combination with ethanol, on the ventricular myocardium of rats. The morphological and ultrastructural alterations induced by the ED in the cardiac muscle, such as enlarged intermyofibrillar spaces and disrupted cristae of mitochondria, were very similar to those produced by alcohol consumption. Those serious ultrastructural modifications were associated with the onset of alcoholic cardiomyopathy.

Overall, our results suggest that the pressor effects of chronic RB treatment would seem to be due to vascular effects rather than increased cardiac contractility. In the future, it will be important to extend the duration of further studies to provide more information on the long-term effects of the use of RB and to better identify possible myocardial dysfunction.

## 4. Materials and Methods

### 4.1. Animals

Thirty-two male Sprague–Dawley rats (Envigo Italy, Udine, Italy) weighing 75 g (28 days old) were housed in the animal facility and given standard food (MIL topi e ratti, GLP diets, Stefano Morini, S. Polo D’Enza, RE, Italy) and water ad libitum. Animals were housed under constant temperature (23 °C), humidity (60%), and a 12 h light/dark cycle (light from 8.00 a.m. to 8.00 p.m.) for 2 weeks.

All animal experiments were conducted in accordance with the guidelines for the care and use of experimental animals of the European Communities Council (2010/63/UE L 276 20/10/2010) and with Italian law (DL: 04.03.2014, N° 26). The study was approved by the organization for animal care of the University of Cagliari (OPBA) and by the Ministero della Salute (authorization n◦ 1152/2015-PR). All surgery was performed under deep anesthesia, and all efforts were made to minimize suffering and the number of animals used. The present research complies with the commonly accepted ‘3Rs’.

### 4.2. Red Bull

RB (100 mL) contains 88.45 g of water, 400 mg of taurine, 32 mg of caffeine, 11 g of carbohydrates, 0.1 g of salt, 8 mg of niacin, 2 mg of pantothenic acid, 2 mg of vitamin B6, and 2 µg of vitamin B12, with a caloric value of 45 kCal [65,79,80].

#### Red Bull Administration

After 2 weeks, adolescent rats aged 42 days, corresponding to approximately 16 years in humans [81], were moved from the animal facility to the treatment room and were assigned randomly to the RB-treated group (*n* = 16) or control group (*n* = 6). To obtain reference values for the amount of fluid intake, the week before the RB treatment (reference week, R Week), water consumption was recorded.

In order to obtain a high consumption of RB in adolescent rats, treated animals received, for 4 weeks, a bottle containing RB from 8:00 p.m. to 8:00 a.m. without access to water. For the same period, the control animals received, from 8:00 p.m. to 8:00 a.m., a bottle filled with water. At 8:00 a.m., the bottles were weighed to measure the consumption of RB and water during the dark phase. All animals received a bottle containing water from 8:00 a.m. to 8:00 p.m. At 8:00 p.m., the bottles were weighed to record the consumption of water during the light phase.

This protocol allowed us to obtain a Red Bull consumption of 320 mL/week, an amount comparable to that reported in young humans by Degirmenci et al. [2]. They reported the amount of Red Bull consumed by them in cans. One can is 250 mL. A total of 4222 subjects consumed at least one can/week, 2576 consumed at least two cans/week, 1289 consumed at least three cans/week, and 1101 consumed at least four cans/week. The consumption in mL was between 250 mL and 1000 mL or more every week. The amount of Red Bull taken weekly by rats is comparable with the amount taken by high consumers (at least four cans).

To obtain reference values, during the R week and the 4 weeks of treatment, the amount of food eaten, the weights, and glycemia of the animals were recorded. Blood glucose content was tested by means of specific strips used on the ONETOUCH Verio Flex (Life Scan).

At the end of the 4 weeks of treatment, rats were anesthetized and unilaterally implanted with a microdialysis probe in the shell or in the core of the NAc or in the mPFCX. In the same surgery session, an intraoral catheter was inserted.

**Figure 7:**
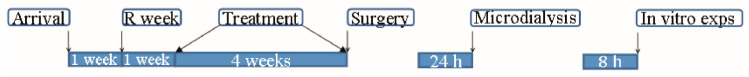
Experimental protocol. The diagram shows the various phases of the experimental protocol.

Figure 7 reports the schematic sequence of experimental events.

### 4.3. Hemodynamic Measurements

Systolic (SPB) and diastolic (DBP) blood pressure and heart rate were measured in conscious rats, using a noninvasive tail-cuff method [82,83,84] (BP Recorder 58500; Ugo Basile, Gemonio (VA), Italy). The sequence of the experimental procedure is described below.

Over the 2 weeks preceding RB intake, rats were habituated extensively to handling and to the following sequences of the experimental procedure:

(1) Habituation to the restraint procedure.
  (1a) Habituation period outside the heated chamber: the rats were immobilized in a plastic holder (6 × 20 × 6 (h) cm) and kept at room temperature for 10 min.
  (1b) Habituation period inside the heated chamber: the rats, immobilized in a plastic holder, were moved to the heated chamber (with the temperature kept at 38 °C); a period of acclimatization to the heated chamber of approximately 10 min was observed before the blood pressure recording was started.
(2) Habituation to the pneumatic pulse sensor chamber: a cuff with a pneumatic pulse sensor was attached to the tail of each rat at the beginning of the 10 min habituation period inside the heated chamber.

Weekly (once a week, every Monday), SBP, DBP, and heart rate were recorded before treatment in each animal by means of a noninvasive tail-cuff recorder, in order to obtain the reference values, and for 4 weeks (weeks I, II, III, and IV) during the treatment and 2 h after bottle removal (RB or water). At each recording, at least three consecutive readings were obtained from each rat, and their average provided the blood pressure and heart rate values of that rat.

Immediately after recording, rats were freed from the holder and returned to their home cages. Furthermore, once a week, every Thursday, the sequence of accustoming the rats to the procedure was repeated (restraint, heated chamber, pneumatic pulse sensor). We measured the blood pressures weekly, as the evaluation of the blood pressure levels every 24 h, usually performed in humans [85], is not applicable in rats.

Moreover, the double product (DP) was calculated at rest, as heart rate × SBP, in order to obtain valuable information about the left-ventricular oxygen consumption, of which the DP is an indirect indicator [86].

### 4.4. Surgery

At the end of the 4 weeks of treatment, under deep anesthesia, as reported by Bassareo et al. [30], a vertical microdialysis probe was stereotaxically and unilaterally implanted, randomly in the left or right hemisphere, using the following coordinates: NAc shell: AP: 2.0 mm and ML: 1 mm from bregma and DV: −7.6 mm from dura; NAc core: AP: 1.6 mm and ML: 1.9 mm from bregma and DV: −7.4 mm from dura; mPFCX: AP: 3.7 mm and ML: 0.8 mm from bregma and DV: −4.8 mm from dura [87]. During the same surgery session, a polyethylene intraoral catheter was implanted at the level of the first molar. The PE tubing passed along the skull and exited near the ear.

After surgery, rats were housed in individual hemispheric cages under the same standard animal facility conditions and were left to recover for at least 24 h. Standard food and water were available ad libitum and were removed before the microdialysis experiment.

### 4.5. Microdialysis

#### 4.5.1. Probe and Oral Catheter Preparation

Microdialysis probes, with a dialyzing portion of 1.5 mm for NAc and 3.0 mm for mPFCX, were prepared with AN69 fibers (HospalDasco, Bologna, Italy), according to the method of Di Chiara et al. [88] as modified by Tanda et al. [89].

Oral catheters were made of a 22-gauge stainless-steel needle and polyethylene (PE) tubing (Polyethylene tubing; Portex limited, Hythe, Kent, UK) (inner diameter 0.58 mm, outer diameter 0.96 mm). The 22-gauge stainless-steel needle was cut on one side, blunted, and inserted in the PE tubing, which ended with a perforated circular disc.

#### 4.5.2. Microdialysis Experiments

Twenty-four hours after the microdialysis probe implantation, the microdialysis experiment was performed. At the beginning of the session, the probes were connected to an infusion pump and perfused with Ringer’s solution (147 mM NaCl, 4 mM KCl, 2.2 mM CaCl2; the use of 2.2 mM Ca^2+^ in the Ringer’s solution can be referenced to the study of Lecca et al. [35]) at a constant rate of 1 µL/min.

After 10 min of probe perfusion by Ringer, dialysate samples (5 for NAc and 10 µL for mPFCX) were collected every 5 or 10 min and injected without further purification into a high-performance liquid chromatograph (HPLC) and an ultra-high-performance liquid chromatograph (UHPLC).

HPLC was equipped with a reverse-phase column (LC-18 DB, 15 cm, 5 µm particle size, Supelco) and a coulometric detector (ESA, Coulochem II, Bedford, MA) to quantify DA. The first and second electrodes of the detector were set at +125 (oxidation) and −175 mV (reduction). The mobile phase comprised 50 mM NaH_2_PO_4_, 0.1 mM Na_2_–EDTA, 0.5 mM *n*-octyl sodium sulfate, and 15% (*v*/*v*) methanol and had a pH of 5.5 (obtained by adding Na_2_HPO_4_). Under these conditions, the sensitivity of the assay for DA was 5 fmol/sample.

UHPLC (ALEXYS Neurotransmitter analyzer, Antec) was equipped with a NeuroSep (C18 110, 1.0 × 100 mm^2^, 1.7 μm) column and an electrochemical amperometric detector (DECADE II SCC). The mobile phase comprised 100 mM phosphoric acid, 100 mM citric acid, 0.1 mM EDTA, and 3 mM acetonitrile (8% *v*/*v*). Under these conditions, the sensitivity of the assay for DA was 5 fmol/sample.

Basal dialysate DA was calculated as the mean of the last three consecutive samples, which differed by no more than 10% and were collected before the experimental session. When basal dialysate DA levels were stable, 2 mL (flow rate 0.2 mL/min) of RB was infused through the oral catheter.

The second RB administration was carried out when the DA concentration returned to the basal level and at least 2 h after the first one.

During RB infusion, the animals’ taste reactivity was recorded. The taste reactivity test was proposed by Grill and Norgren [90] to estimate the valence (positive or negative) and the hedonic impact of taste. The oral catheter was connected to an infusion pump, and the RB was pumped at a constant rate of 0.2 mL/min, for a total amount of 2 mL. During the RB oral infusion, three different kind of taste reactivity reactions were observed: positive, aversive, and neutral reactions. The movements detected for the positive reactions were anterior and lateral tongue protrusion and paw licks, while those for negative ones were forelimb flails, paw tread, gapes, and chin rub, and those for neutral ones were passive solution intake and rhythmic mouth movements [90,91]. The following scoring system was used: one point for each reaction countable as a single event, such as chin rub, paw tread, or gape; for uncountable reactions, such as locomotion and passive solution intake, one point for event that lasts from 1 to 5 s and two points if the event lasts more than 5 s.

At the end of each microdialysis session, rats were sacrificed by decapitation under anesthesia [30]. The brain and heart were removed for histological investigation and cardiac mechanical experiments, respectively.

### 4.6. Histology

**Figure 8:**
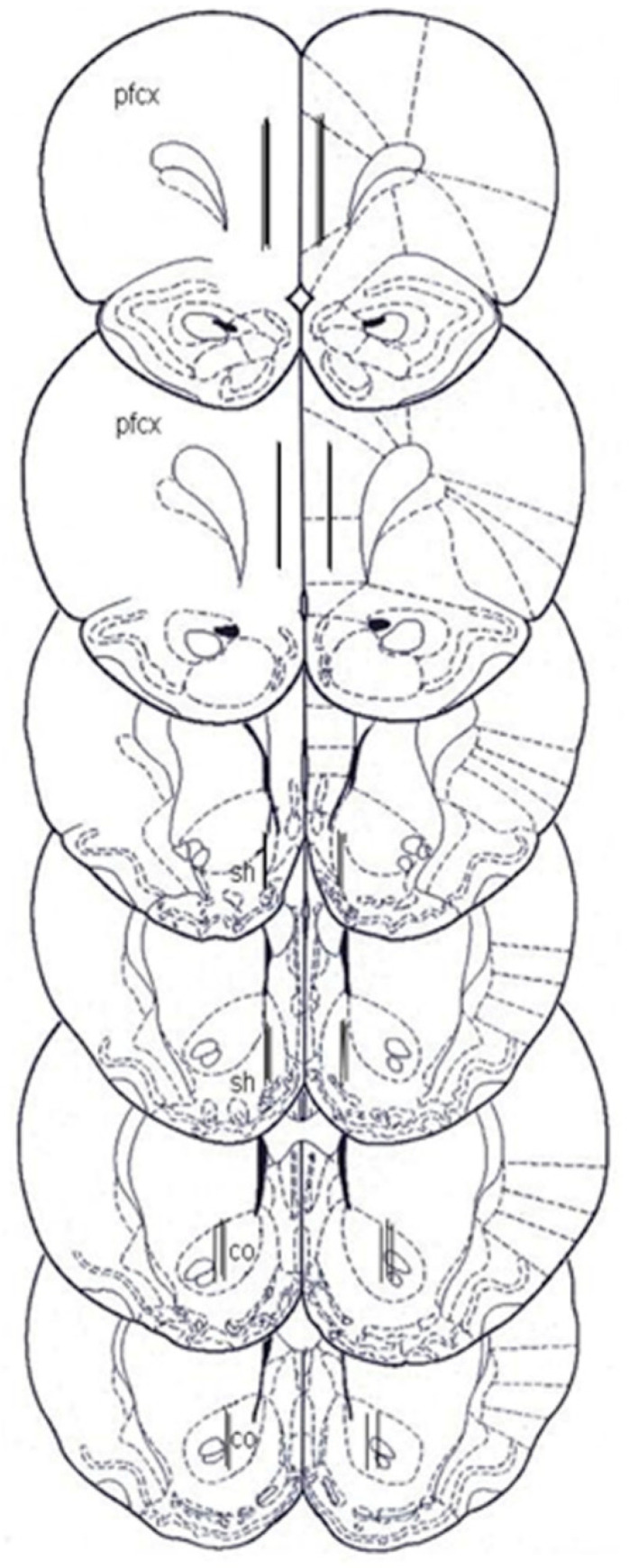
Localization of dialysis probes. Dialysis portions within the mPFCX, NAc shell and core (Adapted from the ref. [87]). pfcx, medial prefrontal cortex; sh, shell; co, core.

The brains, previously removed and postfixed for 5 days, were cut into 100 μm thick serial coronal slices using a Vibratome (Technical Products International, Saint Louis, MO, USA) to establish the location of the dialysis probes. The location of the probes was reconstructed and referred to the atlas of Paxinos and Watson [87] (Figure 8).

### 4.7. Physiology General Procedures

#### 4.7.1. Papillary Muscle Preparation

After decapitation, the hearts were quickly removed and immersed in modified oxygenated Krebs–Hanseleit solution at room temperature, 24 °C. Under stereomicroscope control, the left-ventricle papillary muscles were dissected free.

The experimental arrangement was described previously [92,93,94]. LVPM ends were clamped with two tiny metallic clips (Fine ScienceTools, Vancouver, Canada) and suspended vertically in a 15 mL organ bath containing saline solution with the following composition (in mM): NaCl 123, KCl 6.0, CaCl_2_ 2.50, MgSO_4_ 1.2, NaHCO_3_ 20, KH_2_PO_4_ 1.2, and glucose 11. The bath was aerated vigorously with an O_2_ (95%) and CO_2_ (5%) mixture that maintained a pH of 7.4 at a temperature of 32 °C. For isometric and isotonic studies, the clip on the upper tendinous extremity of the muscle was hanging from a force transducer (Mod.Wp1 Fort 10, 2200 μV/V/g, ADInstrument, Australia) mounted on a rack and pinion, allowing the muscle to be stretched to any desired length and held there isometrically. The lower clip was fixed to the lever of a linear isotonic transducer (moment of inertia, 35 g · cm^−2^; breakaway torque, <0.1 g · cm^−1^; Basile Comerio, Italy). Lever arm loading was equipped with a tungsten alloy cylinder counterweight moving alongside a scale producing a load variation of 0.01 g/step. Two stops enabled the muscle to perform under isometric or isotonic conditions and to receive both preload and afterload. After an equilibration period of 1 h under a preload of 20 mN, LVPM was stimulated supramaximally through platinum plate electrodes placed near the tissue. Electrical stimulation was supplied by a constant-current source (Multiplexing Pulse Booster, Basile, Comerio, Italy) at the optimal force–frequency response for PM, that is, at a frequency of 0.06 Hz and with a stimulus duration of 5 ms. The electrical current intensity was set 10% higher than the minimum necessary to produce a mechanical response (80–100 mA). Experiments were carried out at Lmax, which corresponded to the resting length at the apex of the length–tension curve.

Two signals, force and length, were recorded under isometric or isotonic conditions in control and RB-treated PMs. Under isometric conditions, stimulus responses and length–tension studies were carried out to measure the maximum isometric active developed tension. Under isotonic conditions, both force and shortening signals were simultaneously recorded at preload, corresponding to the passive tension recorded at Lmax, and at various afterloads from preload until the isometric condition was reached. At the end of the experiments, cross-sectional area (CSA, in mm^2^) was calculated from the ratio of fresh PM weight to Lmax, assuming the geometry of a cylinder and a muscle density of 1.056. Force and length data were sampled at a rate of 1 kHz and stored on disc for later analysis. Experimental data were analyzed by using the software Chart V.7.0 equipped with an analog-to-digital converter program (PowerLab, ADInstruments, Australia). A program was projected in our laboratory to calculate the mechanical and energetic parameters of muscle specimens.

#### 4.7.2. Papillary Muscle Mechanical and Energetic Parameters

Under isometric conditions, the following mechanical parameters were measured: maximum developed tension (DT), i.e., the active tension recorded at Lmax, corresponding to peak isometric tension; resting tension (RT), i.e., the passive tension recorded at Lmax; time to peak tension (TPT), the time from the start of the contraction wave to peak tension; half-time of relaxation (T½R), the time needed for the force to fall from its maximum value to half that value; peak rate of tension rise (+T’) and peak rate of tension fall (−T’), being the positive peak rate of the isometric tension derivative and the peak rate of tension decline, respectively. Furthermore, under isotonic conditions, direct evaluations of shortening at preload and at various afterloads were recorded from control and RB-treated LVPMs. DT was normalized per CSA to have the peak isometric tension (P_0_; mN·mm^−2^), as well as RT (mN·mm^−2^). The maximum extent of muscle shortening recorded at preload (ΔL) was expressed as a percentage of Lmax (L/Lmax).

Force–velocity response: the force–velocity relationship was established from the peak shortening velocity (V) plotted against the peak isotonic tension normalized per CSA (P), measured contractions in which afterloads were progressively increased from zero up to the peak isometric tension (P_0_). The maximum unloaded shortening velocity (Vmax) was computed by means of Hill’s equations [95,96]: (P + a) (V + b) = (P_0_ + a) b, where a and b represent the asymptotes of the hyperbola, and P_0_ is the peak isometric tension for V = 0. The shortening velocity was normalized to Lmax. For each LVPM, the F–V relationship was fitted accurately to a hyperbola (each *r* > 0.98).

#### 4.7.3. Crossbridge Characteristics

Huxley’s mathematical model [97] provides an informative system for assessing the number (*ψ* × 10^9^), unitary force (Π_0_), and kinetics of acto-myosin crossbridge (CB) in living muscles. Huxley’s formalism takes into account both force–velocity and heat production for many macroscopic hallmarks of muscle, and it also relates these characteristics to its structural and biochemical properties. By using mechanical parameters, such as shortening velocity, length, and developed force of the entire muscle at various load levels, Huxley’s theory infers the kinetics and number of CBs [97,98]. Considering that the muscle force depends on the unitary CB force and the total number of CBs [97] and the constants of Huxley’s equation parameters h (crossbridge step size equal to 11 nm), e (free energy required to split one ATP molecule, equal to 5.1 × 10^−20^ J), l (the distance between two actin sites, equal to 36 nm), and w (the maximum mechanical work of a unitary crossbridge, equal to 3.8 × 10−20 J), it is possible to estimate the peak values of the rate constants for CB detachment g1 and g2 (s^−1^) and for CB attachment f1 (s^−1^).

$$g2=\frac{2V_{\max}}{h}$$

$$g1=\frac{2\mathrm{wb}}{\mathrm{ehG}}$$

$$f1=\frac{-g1+\sqrt{g1^{2}+4g1g2}}{2}$$

This approach allows us to calculate the unitary force per CB (II_0_, pN) and the number of CB (*Ψ* × 10^9^) per mm^2^ at P_0_.

$$\Pi_{0}=\frac{w}{l}\times \frac{f1}{f1+g1}$$

$$\Psi =\frac{\mathrm{ab}}{e\frac{h}{2l}}\times \frac{f1g1}{f1+g1}.$$

### 4.8. Statistics

Statistical analysis of neurochemical, behavioral, and metabolic data was carried out using Statistica for Windows. Basal dialysate DA was calculated as the mean of the last three consecutive samples differing by no more than 10%. Changes in dialysate DA were expressed as a percentage of the respective baseline values and analyzed by three-way or four-way ANOVA for repeated measures. The number of hedonic and aversive reactions monitored during the RB infusion were analyzed by two-way ANOVA, with previous assessment of the normal distribution. The intake of RB and water, the amount of food eaten, rat body weight, and glycemia blood level data were analyzed by one- or two-way ANOVA. Results showing significant overall changes were subjected to Tukey’s post hoc test, with *p* < 0.05 as the threshold for statistical significance. Hemodynamic data were analyzed by GraphPad Prism 5. Results showing significant overall changes were subjected to Sidak’s post hoc test. A value of *p* < 0.05 was considered to indicate statistical significance. Statistical evaluation of physiological data was performed by applying Student’s *t*-test on selected pairs with OriginPro 7.5 statistics software (Origin Lab Corporation, Northampton, MA, USA), with prior assessment for the normal distribution. A value of *p* < 0.05 was considered to indicate statistical significance.

## 5. Conclusions

We can conclude that chronic RB consumption, particularly during adolescence, modifies the pattern of NAc shell DA responsiveness to RB, making it similar to that observed after drugs of abuse administration. As previously reported by Di Chiara [42,43], the continuous stimulation of DA transmission in this area can facilitate an abnormal association between the primary rewarding stimuli and the neutral stimuli or contexts predictive of them, making the individual prone to attribute an excessive motivational value to reward-associated stimuli. In their presence, individuals can exhibit craving and compulsive seeking of rewarding stimuli. This mechanism can explain the large consumption and then the abuse of EDs taken by different categories of people, such as high-school and university students, athletes, and night workers who need to be awake and stay focused for many hours. They consume EDs without taking into account the negative effects on the cerebral and cardiovascular systems. Furthermore, EDs are usually mixed with spirits and fruit juice to obtain different cocktails. As mentioned above, these associations can amplify the negative effects of EDs and ethanol on the CNS and cardiovascular system and can potentiate their capability to induce addiction. Moreover, heavy ED consumption, as reported by other authors, may be an early precursor to the escalation of substances of abuse [99,100,101]. Further studies are needed to investigate the DA responsiveness during the consumption of EDs and ethanol, in order to clarify if this mix can differentially affect DA transmission in the NAc shell. It will be important to extend this study to female rats, to assess important information on the gender differences.

Long-term treatment with RB from adolescence to adulthood induced changes in hemodynamic parameters and cardiac performance that could be associated with an early impairment of cardiac contractility. These results allow us to hypothesize that chronic consumption of RB during adolescence would lead to a greater likelihood of onset of cardiomyopathy in adulthood.

## Figures and Tables

**Table 1:** Total 24 h fluid consumption expressed in mL in control and RB-treated rats.

Groups	R Week	I Week	II Week	III Week	IV Week
Controls	197.23 ± 7.81	231.79 ± 10.14	243.90 ± 10.88	256.23 ± 12.18	274.91 ± 13.17
RB-treated	205 ± 6.98	319.12 ± 14.85 *	368.60 ± 12.27 *	395.66 ± 27.71 *	434.40 ± 31.4 *

Values are expressed as the mean ± SEM. * *p* < 0.001 with respect to controls.

**Table 2:** Weekly intake of the active ingredients contained in the RB energy drink.

Ingredients	I Week	II Week	III Week	IV Week
Caffeine, mg/pro die	11.87 ± 0.37	14.32 ± 0.43	15.24 ± 0.51	17.28 ± 0.75
Taurine, mg/pro die	148.32 ± 4.66	179.02 ± 5.44	190.50 ± 6.49	216.04 ± 9.40
Sugars, mg/prodie	407.88 ± 12.81	492.30 ± 14.96	523.88 ± 17.84	594.11 ± 25.84
Energy, kcal	14.54 ± 0.79	17.18 ± 0.89	16.89 ± 0.79	21.75 ± 1.83

Values are expressed as the mean ± SEM.

**Table 3:** Isometric and isotonic contraction parameters in control and RB-treated rats.

Parameters	Controls	RB-Treated
**RT**, mN × mm^−2^	9.83 ± 1.01	13.85 ± 3.35
**P_0_**, mN × mm^−2^	37.73 ± 6.24	39.83 ± 5.44
**ΔL**, L/L_max_	0.09 ± 0.01	0.11 ± 0.01
**V_max_**, L_max_ × s^−1^	0.98 ± 0.08	0.97 ± 0.09

Isometric parameters: RT, resting tension, and P_0_, peak isometric tension, normalized per CSA. Isotonic parameters: ΔL, maximum extent of shortening, expressed as a percentage of Lmax, and Vmax, maximum unload shortening velocity, normalized to Lmax. Values are expressed as the mean ± SEM.

## Data Availability

Data is contained within the article.

## References

[1] Reissig C.J., Strain E.C., Griffiths R.R. (2009). Caffeinated energy drinks a growing problem. Drug Alcohol Depend..

[2] Degirmenci N., Fossum I.N., Strand T.A., Vaktskjold A., Holten-Andersen M.N. (2018). Consumption of energy drinks among adolescents in Norway: A cross-sectional study. BMC Public Health.

[3] Mansour B., Amarah W., Nasralla E., Nael E. (2019). Energy drinks in children and adolescents: Demographic data and immediate effects. Eur. J. Pediatr..

[4] Aranda M., Morlock G. (2006). Simultaneous determination of riboflavin, pyridoxine, nicotinamide, caffeine and taurine in energy drinks by planar chromatography-multiple detection with confirmation by electrospray ionization mass spectrometry. J. Chromatogr. A.

[5] Iyadurai S., Chung S. (2007). New-onset seizures in adults: Possible association with consumption of popular energy drinks. Epilepsy Behav..

[6] Ruiz L.D., Scherr R.E. (2018). Risk of Energy Drink Consumption to Adolescent Health. Am. J. Lifestyle Med..

[7] Temple J.L. (2019). Review: Trends, Safety, and Recommendations for Caffeine Use in Children and Adolescents. J. Am. Acad. Child Adolesc. Psychiatry.

[8] Worrall B.B., Phillips C.D., Henderson K.K. (2005). Herbal energy drinks, phenylpropanoid compounds, and cerebral vasculopathy. Neurology.

[9] Machado-Vieira R., Viale C.I., Kapczinski F. (2001). Mania associated with an energy drink: The possible role of caffeine, taurine, and inositol. Can. J. Psychiatry.

[10] Quadri S., Harding L., Lillig M. (2018). An Energy Drink–Induced Manic Episode in an Adolescent. Prim. Care Companion CNS Disord..

[11] Calabrò R.S., Italiano D., Gervasi G., Bramanti P. (2012). Single tonic–clonic seizure after energy drink abuse. Epilepsy Behav..

[12] Wilson R.E., Kado H.S., Samson R., Miller A.B. (2012). A case of caffeine-induced coronary artery vasospasm of a 17-year-old male. Cardiovasc. Toxicol..

[13] Berger A.J., Alford K. (2009). Cardiac arrest in a young man following excess consumption of caffeinated “energy drinks”. Med. J. Aust..

[14] Lippi G., Cervellin G., Sanchis-Gomar F. (2016). Energy drinks and myocardial ischemia: A review of case reports. Cardiovasc. Toxicol..

[15] Dikici S., Saritas A., Besir F.H., Tasci A.H., Kandiset H. (2013). Do energy drinks cause epileptic seizure and ischemic stroke?. Am. J. Emerg. Med..

[16] Grasser E.K., Yepuri G., Dulloo A.G., Montani J.P. (2014). Cardio- and cerebrovascular responses to the energy drink Red Bull in young adults: A randomized cross-over study. Eur. J. Nutr..

[17] Basrai M., Schweinlin A., Menzel J., Mielke H., Weikert C., Dusemund B., Putze K., Watzl B., Lampen A., Bischoff S.C. (2019). Energy Drinks Induce Acute Cardiovascular and Metabolic Changes Pointing to Potential Risks for Young Adults: A Randomized Controlled Trial. J. Nutr..

[18] Ebuehi O.A., Ajayi O.E., Onyeulor A.L., Awelimobor D. (2011). Effects of oral administration of energy drinks on blood chemistry, tissue histology and brain acetylcholine in rabbits. Niger. Q. J. Hosp. Med..

[19] Munteanu C., Rosioru C., Tarba C., Lang C. (2018). Long-term consumption of energy drinks induces biochemical and ultrastructural alterations in the heart muscle. Anatol. J. Cardiol..

[20] Diaz A., Treviño S., Guevara J., Muñoz-Arenas G., Brambila E., Espinosa B., Moreno-Rodríguez A., Lopez-Lopez G., Peña-Rosas U., Venegas B. (2016). Energy Drink Administration in Combination with Alcohol Causes an Inflammatory Response and Oxidative Stress in the Hippocampus and Temporal Cortex of Rats. Oxidative Med. Cell. Longev..

[21] Di Chiara G., Bassareo V. (2007). Reward system and addiction: What dopamine does and doesn’t do. Curr. Opin. Pharmacol..

[22] Bassareo V., Di Chiara G. (1997). Differential influence of associative and nonassociative learning mechanisms on the responsiveness of prefrontal and accumbal dopamine transmission to food stimuli in rats fed ad libitum. J. Neurosci..

[23] Bassareo V., Di Chiara G. (1999). Differential responsiveness of dopamine transmission to food-stimuli in nucleus accumbens shell/core compartments. Neuroscience.

[24] Bassareo V., Di Chiara G. (1999). Modulation of feeding-induced activation of mesolimbic dopamine transmission by appetitive stimuli and its relation to motivational state. Eur. J. Neurosci..

[25] Bassareo V., De Luca M.A., Aresu M., Aste A., Ariu T., Di Chiara G. (2003). Differential adaptive properties of accumbens shell dopamine responses to ethanol as a drug and as a motivational stimulus. Eur. J. Neurosci..

[26] Gambarana C., Masi F., Leggio B., Grappi S., Nanni G., Scheggi S., De Montis M.G., Tagliamonte A. (2003). Acquisition of a palatable-food-sustained appetitive behavior in satiated rats is dependent on the dopaminergic response to this food in limbic areas. Neuroscience.

[27] Rada P., Avena N.M., Hoebel B.G. (2005). Daily bingeing on sugar repeatedly releases dopamine in the accumbens shell. Neuroscience.

[28] Danielli B., Scheggi S., Grappi S., Marchese G., De Montis M.G., Tagliamonte A., Gambarana C. (2010). Modifications in DARPP-32 phosphorylation pattern after repeated palatable food consumption undergo rapid habituation in the nucleus accumbens shell of non-food-deprived rats. J. Neurochem..

[29] Bassareo V., Cucca F., Musio P., Lecca D., Frau R., Di Chiara G. (2015). Nucleus accumbens shell and core dopamine responsiveness to sucrose in rats: Role of response contingency and discriminative/conditioned cues. Eur. J. Neurosci..

[30] Bassareo V., Cucca F., Frau R., Di Chiara G. (2015). Monitoring dopamine transmission in the rat nucleus accumbens shell and core during acquisition of nose-poking for sucrose. Behav. Brain Res..

[31] Bassareo V., Cucca F., Frau R., Di Chiara G. (2015). Differential activation of accumbens shell and core dopamine by sucrose reinforcement with nose poking and with lever pressing. Behav. Brain Res..

[32] Pontieri F.E., Tanda G., Di Chiara G. (1995). Intravenous cocaine, morphine, and amphetamine preferentially increase extracellular dopamine in the ‘‘shell’’ as compared with the ‘‘core’’ of the rat nucleus accumbens. Proc. Natl. Acad. Sci. USA.

[33] Pontieri F.E., Tanda G., Orzi F., Di Chiara G. (1996). Effects of nicotine on the nucleus accumbens and similarity to those of addictive drugs. Nature.

[34] Tanda G., Pontieri F.E., Di Chiara G. (1997). Cannabinoid and heroin activation of mesolimbic dopamine transmission by a common m1 opioid receptor mechanism. Science.

[35] Lecca D., Cacciapaglia F., Valentini V., Di Chiara G. (2006). Monitoring extracellular dopamine in the rat nucleus accumbens shell and core during acquisition and maintenance of intravenous WIN 55,212–2 self-administration. Psychopharmacology.

[36] Lecca D., Cacciapaglia F., Valentini V., Gronli J., Spiga S., Di Chiara G. (2006). Preferential increase of extracellular dopamine in the rat nucleus accumbens shell as compared to that in the core during acquisition and maintenance of intravenous nicotine self-administration. Psychopharmacology.

[37] Lecca D., Cacciapaglia F., Valentini V., Acquas E., Di Chiara G. (2007). Differential neurochemical and behavioral adaptation to cocaine after response contingent and noncontingent exposure in the rat. Psychopharmacology.

[38] Lecca D., Valentini V., Cacciapaglia F., Acquas E., Di Chiara G. (2007). Reciprocal effects of response contingent and noncontingent intravenous heroin on in vivo nucleus accumbens shell versus core dopamine in the rat: A repeated sampling microdialysis study. Psychopharmacology.

[39] Aragona B.J., Cleaveland N.A., Stuber G.D., Day J.J., Carelli R.M., Wightman R.M. (2008). Preferential enhancement of dopamine transmission within the nucleus accumbens shell by cocaine is attributable to a direct increase in phasic dopamine release events. J. Neurosci..

[40] Wiss D.A., Avena N., Rada P. (2018). Sugar. Addiction: From Evolution to Revolution. Front. Psychiatry.

[41] Bassareo V., Cucca F., Frau R., Di Chiara G. (2017). Changes in Dopamine Transmission in the Nucleus Accumbens Shell and Core during Ethanol and Sucrose Self-Administration. Front. Behav. Neurosci..

[42] Di Chiara G. (1998). A motivational learning hypothesis of the role of mesolimbic dopamine in compulsive drug use. J. Psychopharmacol..

[43] Di Chiara G. (1999). Drug addiction as dopamine-dependent associative learning disorder. Eur. J. Pharmacol..

[44] Acquas E., Tanda G., Di Chiara G. (2002). Differential effects of caffeine on dopamine and acetylcholine transmission in brain areas of drug-naive and caffeine-pretreated rats. Neuropsychopharmacology.

[45] De Luca M.A., Bassareo V., Bauer A., Di Chiara G. (2007). Caffeine and accumbens shell dopamine. J. Neurochem..

[46] Solinas M., Ferre S., You Z.B., Karcz-Kubicha M., Popoli P., Goldberg S.R. (2002). Caffeine induces dopamine and glutamate release in the shell of the nucleus accumbens. J. Neurosci..

[47] Quarta D., Ferre S., Solinas M., You Z.B., Hockemeyer J., Popoli P., Goldberg S.R. (2004). Opposite modulatory roles for adenosine A1 and A2A receptors on glutamate and dopamine release in the shell of the nucleus accumbens. Effects of chronic caffeine exposure. J. Neurochem..

[48] Galvalisi M., Prieto J.P., Martínez M., Abin-Carriquiry J.A., Scorza C. (2017). Caffeine Induces a Stimulant Effect and Increases Dopamine Release in the Nucleus Accumbens Shell Through the Pulmonary Inhalation Route of Administration in Rats. Neurotox. Res..

[49] Cacciapaglia F., Saddoris M.P., Wightman R., Carelli R.M. (2012). Differential dopamine release dynamics in the nucleus accumbens core and shell track distinct aspects of goal-directed behavior for sucrose. Neuropharmacology.

[50] Lai F., Cucca F., Frau R., Corrias F., Schlich M., Caboni P., Fadda A.M., Bassareo V. (2018). Systemic Administration of Orexin a Loaded Liposomes Potentiates Nucleus Accumbens Shell Dopamine Release by Sucrose Feeding. Front. Psychiatry.

[51] Hajanal A., Norgren R. (2002). Repeated access to sucrose augments dopamine turnover in the nucleus accumbens. Neuroreport.

[52] Avena N.M., Rada P., Moise N., Hoebel B.G. (2006). Sucrose sham feeding on a binge schedule releases accumbens dopamine repeatedly and eliminates the acetylcholine satiety response. Neuroscience.

[53] Ericsson M., Molander A., Stomberg R., Söderpalm B. (2006). Taurine elevates dopamine levels in the rat nucleus accumbens; antagonism by strychnine. Eur. J. Neurosci..

[54] Zucconi S., Volpato C., Adinolfi F., Gandini E., Gentile E., Loi A., Fioriti L. (2013). Gathering consumption data on specific consumer groups of energy drinks. EFSA Support. Publ..

[55] Christensen L.M., Iversen J.D., Biltoft-Jensen A., Petersen M.A., Søndergaard A.B., Matthiessen J. (2014). Consumption of Energy Drinks among 10–35-yr-old Danes (in Danish with an English Summary).

[56] Visram S., Cheetham M., Riby D.M., Crossley S.J., Lake A.A. (2016). Consumption of energy drinks by children and young people: A rapid review examining evidence of physical effects and consumer attitudes. BMJ Open.

[57] Seifert S.M., Schaechter J.L., Hershorin E.R., Lipshultz S.E. (2011). Health effects of energy drinks on children, adolescents, and young adults. Pediatrics.

[58] Wolk B.J., Ganetsky M., Babu K.M. (2012). Toxicity of energy drinks. Curr. Opin. Pediatr..

[59] Arria A.M., Caldeira K.M., Kasperski S.J., O’Grady K.E., Vincent K.B., Griffiths R.R., Wish E.D. (2010). Increased alcohol consumption, nonmedical prescription drug use, and illicit drug use are associated with energy drink consumption among college students. J. Addict. Med..

[60] Terry-McElrath Y.M., O’Malley P.M., Johnston L.D. (2014). Energy drinks, soft drinks, and substance use among United States secondary school students. J. Addict. Med..

[61] Woolsey C.L., Barnes L.B., Jacobson B.H., Kensinger W.S., Barry A.E., Beck N.C., Resnik A.G., Evans M.W. (2014). Frequency of energy drink use predicts illicit prescription stimulant use. Subst. Abus..

[62] Arria A.M., Caldeira K.M., Bugbee B.A., Vincent K.B., O’Grady K. (2017). Energy drink use trajectories predict substance use outcomes. Drug Alcohol Depend. Coll. Probl. Drug Depend..

[63] Jackson D.B., Leal W.E. (2018). Energy drink consumption and the perceived risk and disapproval of drugs: Monitoring the Future, 2010–2016. Drug Alcohol Depend..

[64] Clark K.S., Coleman C., Shelton R., Heemstra L.A., Novak C.M. (2019). Caffeine enhances activity thermogenesis and energy expenditure in rats. Clin. Exp. Pharmacol. Physiol..

[65] Miles-Chan J.L., Charrière N., Grasser E.K., Montani J.P., Dulloo A.G. (2015). The thermic effect of sugar-free Red Bull: Do the non-caffeine bioactive ingredients in energy drinks play a role?. Obesity.

[66] Acheson K.J., Zahorska-Markiewicz B., Pittet P., Anantharaman K., Jéquier E. (1980). Caffeine and coffee: Their influence on metabolic rate and substrate utilization in normal weight and obese individuals. Am. J. Clin. Nutr..

[67] Dulloo A.G., Seydoux J., Girardier L. (1992). Potentiation of the thermogenic antiobesity effects of ephedrine by dietary methylxanthines: Adenosine antagonism or phosphodiesterase inhibition?. Metabolism.

[68] Novak C.M., Levine J.A. (2007). Central neural and endocrine mechanisms of non-exercise activity thermogenesis and their potential impact on obesity. J. Neuroendocrinol..

[69] Hursel R., Westerterp-Plantenga M.S. (2013). Catechin- and caffeine-rich teas for control of body weight in humans. Am. J. Clin. Nutr..

[70] Bracale R., Petroni M.L., Davinelli S., Bracale U., Scapagnini G., Carruba M.O., Nisoli E. (2014). Muscle Uncoupling Protein 3 Expression Is Unchanged by Chronic Ephedrine/Caffeine Treatment: Results of a Double Blind, Randomised Clinical Trial in Morbidly Obese Females. PLoS ONE.

[71] Miles-Chan J.L., Charrière N., Grasser E.K., Montani J.P., Dulloo A.G. (2015). The blood pressure-elevating effect of Red Bull energy drink is mimicked by caffeine but through different hemodynamic pathways. Physiol. Rep..

[72] Flinn S., Gregory J., McNaughton L.R., Tristram S., Davies P. (1990). Caffeine ingestion prior to incremental cycling to exhaustion in recreational cyclists. Int. J. Sports Med..

[73] Nienhueser J., Brown G.A., Shaw B.S., Shaw I. (2011). Effects of Energy Drinks on Metabolism at Rest and During Submaximal Treadmill Exercise in College Age Males. Int. J. Exerc. Sci..

[74] Franks A.M., Schmidt J.M., McCain K.R., Fraer M. (2012). Comparison of the effects of energy drink versus caffeine supplementation on indices of 24-hour ambulatory blood pressure. Ann. Pharmacother..

[75] Savoca M.R., Evans C.D., Wilson M., Harshfield G.A., Ludwig D.A. (2004). The Association of Caffeinated Beverages with Blood Pressure in adolescent. Arch. Pediatr. Adolesc. Med..

[76] Savoca M.R., MacKey M.L., Evans C.D., Wilson M., Ludwig D.A., Harshfield G.A. (2005). Association of ambulatory blood pressure and dietary caffeine in adolescents. Am. J. Hypertens..

[77] Pincomb G.A., Lovallo W.R., Passey R.B., Whitsett T.L., Silverstein S.M., Wilson M.F. (1985). Effects of caffeine on vascular resistance, cardiac output and myocardial contractility in young men. Am. J. Cardiol..

[78] Sung B.H., Lovallo W.R., Pincomb G.A., Wilson M.F. (1990). Effects of caffeine on blood pressure response during exercise in normotensive healthy young men. Am. J. Cardiol..

[79] Higgins J.P., Tuttle T.D., Higgins C.L. (2010). Energy beverages: Content and safety. Mayo Clin. Proc..

[80] Grasser E.K., Miles-Chan J.L., Charrière N., Loonam C.R., Dulloo A.G., Montani J.P. (2016). Energy Drinks and Their Impact on the Cardiovascular System: Potential Mechanisms. Adv. Nutr..

[81] Spear L.P. (2015). Adolescent alcohol exposure: Are there separable vulnerable periods within adolescence?. Physiol. Behav..

[82] Kurtz T.W., Griffin K.A., Bidani A.K., Davisson R.L., Hall J.E. (2005). Recommendations for blood pressure measurement in animals. Arterioscler. Thromb. Vasc. Biol..

[83] Virdis A., Colucci R., Fornai M., Blandizzi C., Duranti E., Pinto S., Bernardini N., Segnani C., Antonioli L., Taddei S. (2005). Cyclooxygenase-2 Inhibition Improves Vascular Endothelial Dysfunction in a Rat Model of Endotoxic Shock: Role of Inducible Nitric-Oxide Synthase and Oxidative Stress. J. Pharmacol. Exp. Ther..

[84] Mazzone G., Morisco C., Lembo V., D’Argenio G., D’Armiento M., Rossi A., Giudice C.D., Trimarco B., Caporaso N., Morisco F. (2018). Dietary supplementation of vitamin D prevents the development of western diet-induced metabolic, hepatic and cardiovascular abnormalities in rats. United Eur. Gastroenteral J..

[85] Bassareo P.P., Marras A.R., Pasqualucci D., Mercuro G. (2010). Increased arterial rigidity in children affected by Cushing’s syndrome after successful surgical cure. Cardiol. Young.

[86] Van Vliett B.N., Montani J.P. (1999). Baroreflex stabilization of the double product. Am. J. Physiol..

[87] Paxinos G., Watson C. (1998). The Rat Brain in Stereotaxic Coordinates.

[88] Di Chiara G., Tanda G., Frau R., Carboni E. (1993). On the preferential release of dopamine in the nucleus accumbens by amphetamine: Further evidence obtained by vertically implanted concentric dialysis probes. Psychopharmacoly.

[89] Tanda G., Bassareo V., Di Chiara G. (1996). Mianserin markedly and selectively increases extracellular dopamine in the prefrontal cortex as compared to the nucleus accumbens of the rat. Psychopharmacology.

[90] Grill H.J., Norgren R. (1978). The taste reactivity test. I. Mimetic responses to gustatory stimuli in neurologically normal rats. Brain Res..

[91] Berridge K.C., Robinson T.E. (1998). What is the role of dopamine in reward: Hedonic impact, reward learning, or incentive salience?. Brain Res. Rev..

[92] Eisenberg E., Hill T.L., Chen Y.D. (1980). Cross-bridge model of muscle contraction: Quantitative analysis. Biophys. J..

[93] Palmiter K.A., Tyska M.J., Dupuis D.E., Alpert N.R., Warshaw D.M. (1999). Kinetics differences at the single molecule level account for the functional diversity of rabbit cardiac myosin isoforms. J. Physiol..

[94] Vargiu R., Littarru G.P., Fraschini M., Perinu A., Tiano L., Capra A., Mancinelli R. (2010). Enhancement of shortening velocity, power, and acto-myosin cross bridge (CB) kinetics following long-term treatment with propionyl-L-carnitine, coenzyme Q10 and omega-3 fatty acids in BIO T0-2 cardiomyopathic. Syrian Hamsters papillary muscle. BioFactors.

[95] Hill A.V. (1938). The heat of shortening and the dynamic constants of muscle. Proc. R. Soc. Lond. Ser. B Biol. Sci..

[96] Coirault C., Chemla D., Péry-Man N., Suard I., Lecarpentier Y. (1995). Effects of fatigue on tension-velocity relation of diaphragm. Energetic implication. Am. J. Respir. Crit. Care Med..

[97] Huxley A.F., Simmons R.M. (1971). Mechanical properties of the cross-bridges of frog striated muscle. J. Physiol..

[98] Blanc F.X., Coirault C., Salmeros S., Chemla D., Lecarpentier Y. (2003). Mechanics and crossbridge kinetics of tracheal smooth muscle in two inbred rat strains. Eur. Respir. J.

[99] Housman J.M., Williams R.D. (2018). Adolescent Nonmedical Use of Opioids and Alcohol Mixed with Energy Drinks. Am. J. Health Behav..

[100] Leal W.E., Jackson D.B. (2019). The role of energy drink consumption in the intention to initiate marijuana use among adolescents. Addict. Behav..

[101] Marmorstein N.R. (2019). Investigating associations between caffeinated beverage consumption and later alcohol consumption among early adolescents. Addict. Behav..

